# Supplementary material for: The Connectivity of the Resting Brain in Primary Open-Angle Glaucoma: A Systematic Review
Source: Biomedicines. 2025 Jun 7;13(6):1402. doi: 10.3390/biomedicines13061402 (PMC12190858; doi:10.3390/biomedicines13061402)
Supplement: Supplementary file 1 [file biomedicines-13-01402-s001.zip › biomedicines-3570873-supplementary.pdf]

In order to identify records, we searched the 3 databases using the keywords glaucoma and functional magnetic resonance imaging, as well as variations for the latter. We also applied filters against articles written in a language different from English, as well against review papers. Our search queries were as follows:

PUBMED: ("glaucoma") AND ("fMRI" OR "functional MRI" OR "resting state") AND (english[Filter]) NOT review [publication type]

SCOPUS: TITLE-ABS-KEY (“glaucoma”) AND TITLE-ABS-KEY ("fMRI" OR "functional MRI" OR "resting state") AND (LIMIT-TO (LANGUAGE , "English")) AND (EXCLUDE (DOCTYPE , "re"))

WOS: (TS=(glaucoma)) AND TS=(fMRI OR "functional MRI" OR "resting state") + *filter to exclude reviews, include only English*
